# Clinical Validation of Targeted Next Generation Sequencing for Colon and Lung Cancers

**DOI:** 10.1371/journal.pone.0138245

**Published:** 2015-09-14

**Authors:** Nicky D’Haene, Marie Le Mercier, Nancy De Nève, Oriane Blanchard, Mélanie Delaunoy, Hakim El Housni, Barbara Dessars, Pierre Heimann, Myriam Remmelink, Pieter Demetter, Sabine Tejpar, Isabelle Salmon

**Affiliations:** 1 Department of Pathology, Erasme Hospital, Université Libre de Bruxelles, Brussels, Belgium; 2 Department of Genetics, Erasme Hospital,Université Libre de Bruxelles, Brussels, Belgium; 3 Department of Oncology, University Hospital Leuven, Leuven, Belgium; University of Verona, ITALY

## Abstract

**Objective:**

Recently, Next Generation Sequencing (NGS) has begun to supplant other technologies for gene mutation testing that is now required for targeted therapies. However, transfer of NGS technology to clinical daily practice requires validation.

**Methods:**

We validated the Ion Torrent AmpliSeq Colon and Lung cancer panel interrogating 1850 hotspots in 22 genes using the Ion Torrent Personal Genome Machine. First, we used commercial reference standards that carry mutations at defined allelic frequency (AF). Then, 51 colorectal adenocarcinomas (CRC) and 39 non small cell lung carcinomas (NSCLC) were retrospectively analyzed.

**Results:**

Sensitivity and accuracy for detecting variants at an AF >4% was 100% for commercial reference standards. Among the 90 cases, 89 (98.9%) were successfully sequenced. Among the 86 samples for which NGS and the reference test were both informative, 83 showed concordant results between NGS and the reference test; i.e. *KRAS* and *BRAF* for CRC and *EGFR* for NSCLC, with the 3 discordant cases each characterized by an AF <10%.

**Conclusions:**

Overall, the AmpliSeq colon/lung cancer panel was specific and sensitive for mutation analysis of gene panels and can be incorporated into clinical daily practice.

## Introduction

Recent advances in sequencing technology have enabled comprehensive profiling of genetic alterations in cancer [1]. The development of tyrosine kinase inhibitor treatments has made it important to test cancer patients for clinically significant gene mutations that influence the benefit of treatment. Identification of cancer-associated mutations has become standard care for cancer treatment; examples of such include *RAS* mutations in metastatic colorectal carcinomas or *EGFR* mutations in lung cancer. Routine *EGFR* somatic mutation testing is now recommended in Europe and United States for non-squamous non small cell lung carcinomas (NSCLC) [2, 3]. New European guidelines strongly encourage a wide coverage of exons 18–21 [2]. Moreover, new NCCN Guidelines for NSCLC strongly endorses broader molecular profiling with the goal of identifying rare driver mutations for which effective drugs may already be available, or to appropriately counsel patients regarding the availability of clinical trials (NCCN guidelines http://www.nccn.org/professionals/physician_gls/pdf/nscl.pdf). Until recently, indications for standard-of-care molecular testing in colorectal carcinomas included testing for *KRAS* mutational status as a predictor of response to anti–epidermal growth factor receptor (EGFR) agents such as cetuximab [4]. Now, guidelines recommend that at the very least, exon 2 *KRAS* mutation status should be determined and whenever possible, non-exon 2 *KRAS* and *NRAS* mutation statuts should be also determined (NCCN guidelines http://www.nccn.org/professionals/physician_gls/pdf/colon.pdf). This underlines that the number (or the extent) of biomarkers that will be need to be assessed in clinical daily practice in molecular pathology is rapidly increasing. This calls for the implementation of methods probing the mutational status of multiple genes. Moreover, this increase in the number of genes to test is associated with a decrease in the sample size. The pathologist is facing a new challenge: optimization of available tumor tissue. As the number of clinically significant genetic variants has increased, clinical testing has evolved, moving from single mutations to multiplex hotspot evaluations in multiple cancer genes. In recent years, Next Generation Sequencing (NGS) has begun to supplant other technologies for gene mutation testing [5–8]. Targeted, amplicon-based NGS offers simultaneous sequencing of thousands of short DNA sequence in a massively parallel way and may offer a cost effective approach for detecting multiple genetic alterations with a minimum amount of DNA [5, 9, 10]. Moreover, NGS can be performed using DNA from formalin-fixed, paraffin-embedded (FFPE) tissue blocks [11–16]. The clinical application of NGS in cancer is the detection of clinically actionable genetic/genomic alterations that are critical for cancer care [6]. These alterations can be of diagnostic, prognostic, or therapeutic significance. However, transfer of NGS technology to clinical daily practice requires validation.

In the present study we evaluated the clinical applicability of the Ion Ampliseq Colon and Lung cancer panel on the Ion Torrent Personal Genome Machine (PGM—Life Technologies) to screen lung and colorectal cancers. The Ion Ampliseq Colon and Lung cancer panel is a multiplex PCR-based library preparation method by which 90 amplicons that encompass 1825 mutational hotspots of 22 genes related to colon and lung cancer are selectively amplified [14, 15, 17, 18].

## Materials and Methods

### Ethics Statement

This work has been approved by the ethical committee of the Erasme University Hospital (Brussels, Belgium—ref: P2013/174). According to the Belgian law of December 2008 « Loi relative à l'obtention et à l'utilisation de matériel corporel humain destiné à des applications médicales humaines ou à des fins de recherche scientifique », no written informed consent was required. The ethical committee has thus waived the need for written informed consent from the participant.

### Samples selection

Tumor samples from 90 patients were retrospectively analyzed, including 51 colorectal adenocarcinomas (CRC) and 39 non small cell lung carcinomas (NSCLC including 37 adenocarcinomas and 2 squamous carcinomas). The mutational status of *KRAS* and *BRAF* in CRC and of *EGFR* in NSCLC had been assessed previously in the context of daily practice. The primary sample types were either surgical resections (n = 57, 44 CRC and 13 NSCLC), biopsies (n = 23, 7 CRC and 16 NSCLC) or cell blocks (n = 10, all NSCLC). In addition, we used 12 non neoplastic samples (6 lungs and 6 colons) and 5 commercial FFPE reference standards (Horizon Diagnostics, Cambridge, UK) carrying mutation in *NRAS*, *KRAS*, *AKT* and *EGFR* at 50% allelic frequency (AF) and 1 FFPE multiplex reference standard (Horizon Diagnostics, Cambridge, UK) carrying 11 different mutations at various defined AF (from 0.9 to 24.4%).

### DNA extraction

DNA was extracted from FFPE tumor samples using the QIAamp FFPE tissue kit (Qiagen, Antwerp, Belgium). Briefly, unstained 10 μm paraffin sections were cut and incubated at 37°C in a drying oven overnight. The paraffin was removed by incubating the slides in 2 successive baths of xylene and the tumor tissue was manually macrodissected, scraped off the slide with a scalpel and transferred into a 1.5ml tube. DNA was then extracted according to the manufacturer’s instructions. The H&E stained slide from the same block, previously reviewed by a pathologist who circled the tumor area and evaluated the tumor percentage, was used as a guide for the macrodissection. The percentage of tumor cells of the samples ranged from 5 to 90%. The DNA obtained was quantified using the Qubit® fluorometer in combination with the Qubit® dsDNA HS assay kit (Life Technologies, Gent, Belgium).

### Detection of *KRAS*, *BRAF* and *EGFR* mutations

Detection of *KRAS*, *BRAF* and *EGFR* mutations were performed in the context of clinical daily practice in an ISO15189-certified laboratory by quantitative PCR. These methods are described in S1 File. The sensitivity of these assays is varied between 3 and 20% of mutant DNA for the KRAS testing, 10% of mutant DNA for BRAF testing, 0.5% of mutant DNA for EGFR p.L858R testing, 1% of mutant DNA for EGFR exon 19 deletion and 5% of mutant DNA for EGFR p.T790M testing,

### Droplet digital PCR

Some mutations detected by NGS were validated by droplet digital PCR (ddPCR), as detailed in S1 File.

### Next generation sequencing

For library construction, 10 ng of DNA (measured using the Qubit® fluorometer in combination with the Qubit® dsDNA HS assay kit) was amplified using the Colon and Lung Cancer panel (Ampliseq™, Life Technologies), a panel recently validated [15] and the Ion Ampliseq™ HiFi Master Mix (Ion Ampliseq™ Library kit 2.0). An amplicon library was thus generated for sequencing 1825 hotspot mutations in 22 genes including *AKT1 (NM_05163)*, *ALK* (*NM_004304)*, *BRAF (NM_004333)*, *CTNNB1 (NM_001904)*, *DDR2 (*NM_001014796), *EGFR (*NM_005228), *ERBB2 (*NM_004448), *ERBB4 (*NM_005235), *FBXW7 (*NM_033632), *FGFR1 (*NM_023110), *FGFR2 (*NM_022970), *FGFR3 (*NM_000142), *KRAS (*NM_033360), *MAP2K1 (*NM_002755), *MET (*NM_001127500), *NOTCH1 (*NM_017617), *NRAS (*NM_002524), *PIK3CA (*NM_006218), *PTEN (*NM_000314), *SMAD4 (*NM_005359), *STK11 (*NM_000455), *TP53 (*NM_000546). The amplicons were then digested, barcoded and amplified using the Ion Ampliseq™ Library kit 2.0 and Ion Xpress™ barcode adapters kit (Life technologies) according to the manufacturer’s instructions. The library was quantified using the Qubit® fluorometer and the Qubit® dsDNA HS assay kit (Life technologies). 8pM of each library was multiplexed and clonally amplified on Ion sphere™ particles (ISP) by emulsion PCR performed on the Ion One Touch 2 instrument with the Ion PGM™ template OT2 200 kit (Life technologies) according to the manufacturer’s instructions. Quality control was performed using the Ion Sphere™ Quality Control kit (Life Technologies) to ensure that 10–30% of template positive ISP were generated in the emulsion PCR. Finally, the template ISP were enriched, loaded on an Ion 316™ or on an Ion 318™ chip and sequenced on a PGM™ sequencer with the Ion PGM™ sequencing 200 kit v2 according to the manufacturer’s instructions.

### Data Analysis

The raw data were analyzed using the torrent suite software v3.6.2 (Life technologies). The coverage analysis was performed using the coverage analysis plug-in v3.6. Cases for which the number of mapped reads was <100000 and/or the average base coverage was <500x were considered as non informative. Mutations were detected using the Variant Caller plug-in v3.6 with low stringency settings (Life Technologies). In the variant list obtained, each mutation was verified in the Integrative genome viewer (IGV) from the Broad Institute (http://www.broadinstitute.org/igv/) [19]. Only mutations reported in the COSMIC (Sanger Institute Catalogue of Somatic Mutations in Cancer) database (http://www.sanger.ac.uk/cosmic) were taken into account and silent or intronic mutations were not reported.

### Statistical analyses

The non-parametric Mann-Whitney and Kruskal-Wallis tests were used to compare two, or multiple, independent groups of numerical data, respectively. If the Kruskal-Wallis test was significant, post-hoc tests were applied using either the standard Dunn procedure to compare all group pairs or its adaptation to compare each experimental condition to the control, avoiding multiple comparison effects (as detailed in Zar [20]).

All statistical analyses were performed using Statistica (Statsoft, Tulsa, OK, USA) and p-values < 0.05 were considered significant.

## Results

### NGS panel validation

The performance of the AmpliSeq Colon and Lung Cancer panel was first evaluated using 12 non neoplastic tissues (6 lungs and 6 colons) and 6 commercial FFPE reference standards (5 reference standards with one mutation at 50% allelic frequency and one multiplex reference standard carrying 11 different mutations at various defined allelic frequencies, varying from 0.9 to 24.4%).

No mutation was detected in the 12 non neoplastic tissues. The 5 mutations present in the 5 reference standards at 50% allelic frequency were all correctly detected by NGS with the AmpliSeq Colon and Lung Cancer panel (Table 1). Among the 11 mutations present in the multiplex reference standard, all mutations with AF >3% were correctly detected by NGS with the exception of the *KIT* mutation because this gene is not included in the 22 genes of the panel. For the 3 mutations with AF <3%, only one (*EGFR* deletion in exon 19, AF = 2.0%) was detected by the Variant Caller whereas the two others (*EGFR* p.L858R and p.T790M with AF = 2.7% and 0.9% respectively) were not. By IGV inspection, we found that these variants were present but with low AF (25/1633 reads (1.5%) and 7/1613 reads (0.4%), respectively). Additional mutations in *CTNNB1*, *BRAF*, *PIK3CA* and *EGFR* were detected by the Variant Caller in the reference standards (Table 1). The *KRAS* and *NRAS* standards are generated from the SW48 cell line which is reported to carry *CTNNB1* p.S33Y and *EGFR* p.G719S mutations in the COSMIC database (http://www.sanger.ac.uk/cosmic); the *EGFR* standards are generated from the RKO cell line which is reported to carry *BRAF* p.V600E and *PIK3CA* p.H1047R mutations. Finally, the multiplex reference standard is generated from the RKO, SW48 and HCT16 cell lines, which explains the detection of the *CTNNB1* p.S33Y mutation in this control.

**Table 1:** NGS analysis of reference standards.

Sample	Expected mutation	Expected allelic frequency	NGS result	NGS allelic frequency	Concordance
**Reference standard**	*AKT* p.E17K	50%	*AKT* p.E17K	67.0%	Yes
**Reference standard**	*KRAS* p.G12C	50%	*KRAS* p.G12C	41.0%	Yes
			*CTNNB1* p. S33Y	49.3%	SW48 cell line additional mutations
			*EGFR* p.G719S	34.8%	SW48 cell line additional mutations
**Reference standard**	*NRAS* p.Q61L	50%	*NRAS* p.Q61L	56.4%	Yes
			*CTNNB1* p. S33Y	55.5%	SW48 cell line additional mutations
			*EGFR* p.G719S	42%	SW48 cell line additional mutations
**Reference standard**	*EGFR* p.E746-A750delELREA	50%	*EGFR* p.E746-A750delELREA	47.5%	Yes
			*PIK3CA* p.H1047R	48.8%	RKO cell line additional mutations
			*BRAF* p.V600E	65.3%	RKO cell line additional mutations
**Reference standard**	*EGFR* p.L858R	50%	*EGFR* p.L858R	45.6%	Yes
			*PIK3CA* p.H1047R	47.5%	RKO cell line additional mutations
			*BRAF* p.V600E	65.4%	RKO cell line additional mutations
**Multiplex reference standard**	*BRAF* p.V600E	10.2%	*BRAF* p.V600E	8.5%	Yes
	*KIT* p.D816V	10.4%	Not included	NA	NA
	*EGFR* p.E746-A750delELREA	2.0%	*EGFR* p.E746-A750delELREA	2.0%	Yes
	*EGFR* p.L858R	2.7%	Not detected	NA	No
	*EGFR* p.T790M	0.9%	Not detected	NA	No
	*EGFR* p.G719S	24.4%	*EGFR* p.G719S	25.4%	Yes
	*KRAS* p.G13D	16.1%	*KRAS* p.G13D	13.5%	Yes
	*KRAS* p.G12D	5.0%	*KRAS* p.G12D	5.9%	Yes
	*NRAS* p.Q61K	12.8%	*NRAS* p.Q61K	10.7%	Yes
	*PIK3CA* p.H1047R	18.6%	*PIK3CA* p.H1047R	16.0%	Yes
	*PIK3CA* p.E545K	8.9%	*PIK3CA* p.E545K	8.2%	Yes
			*CTNNB1*p. S33Y	35.8%	W48 cell line additional mutation

The precision (reproducibility and repeatability) was also evaluated using 2 FFPE tumour samples and the multiplex reference standard. The samples were analysed 5 times (5 library productions starting from the same DNA extract) in three different experiments (Table 2). All mutations with an AF >4% were consistently detected. However, mutations with an AF <3% were detected by the Variant Caller in one or more, but not all, of the five replicates. By IGV inspection, the TP53 mutations inconsistently detected by the Variant Caller were present only in the replicate for which the mutation was detected by the Variant Caller, but not for the other replicates (S1 Table). For the reference standard, the 3 EGFR variants were detected by IGV inspection (but with a variant coverage < 30x for the majority of the replicates) although these were inconsistently detected by the Variant Caller (S1 Table).

**Table 2:** Precision (reproducibility and repeatability) evaluated for 2 FFPE tumour samples and the multiplex reference standard.

		NGS results						ddPCR results
Sample	Mutations	Rep 1	Rep 2	Rep 3	Rep 4	Rep 5	Mean (SD)	
**1**	*BRAF* p.V600E	9.3%	11.1%	9.7%	12.6%	10.6%	10.6% (1.3)	
	*PIK3CA* p.H1047Q	10.1%	10.2%	9.1%	12.4%	12.4%	10.8% (1.5)	9.1%
	*TP53* p.R181C	1.8%	ND	ND	ND	ND		ND
	*TP53* p.H168Y	ND	ND	ND	1.8%	ND		ND
	*TP53* p.R213Q	ND	2.0%	ND	ND	ND		
**2**	*EGFR* p.L858R	14.0%	11.6%	11.6%	9.8%	12.3%	11.9% (1.5)	
	*EGFR* p.T790M	2.2%	2.5%	2.9%	2.1%	2.7%	2.5% (0.3)	
	*NOTCH1* p.V1758delV	ND	ND	ND	1.7%	ND		
	*TP53* p.H178N	ND	ND	ND	ND	2.0%		
**Multiplex reference standard (mutations with AF <15%)**	*BRAF* p.V600E *(10*.*2%)*	8.5%	9.6%	11.4%	9.6%	10.2%	9.9% (1)	
	*NRAS* p.Q61K *(12*.*8%)*	10.7%	9.4%	8.5%	10.7%	8.6%	9.6% (1.1)	
	*PIK3CA* p.E545K *(8*.*9%)*	8.2%	8.3%	7.3%	8.2%	9.0%	8.2% (0.6)	
	*KRAS* p.G12D *(5*.*0%)*	5.9%	5.4%	5.6%	4.6%	5.8%	5.5% (0.5)	
	*EGFR* p.E746-A750delELREA *(2*.*0%)*	2.0%	ND	ND	2.4%	ND		
	*EGFR* p.L858R *(2*.*7%)*	ND	ND	1.7%	ND	ND		
	*EGFR* p.T790M *(0*.*9%)*	ND	ND	ND	ND	ND		

ND: not detected, Rep: replicate, SD: standard deviation.

Moreover, some mutations were verified by ddPCR (Table 2). The p.H1047Q PIK3CA mutation, consistently detected by NGS with a mean AF of 10.8%, was also detected by ddPCR with an AF of 9.1%. In contrast, the p.R181C and the p.H168Y TP53 mutations inconsistently detected by NGS were not detected by ddPCR. Given the facts that mutations detected with an AF < 3% were not validated by ddPCR (for TP53 mutations) or inconsistently detected (EGFR mutations in the reference standard), and that the KRAS mutation with an expected AF of 5% was consistently detected with an AF varying from 4.6 to 5.9%, we selected a 4% AF threshold for mutation reporting. This threshold is consistent with the data reported in the literature for this NGS platform [9, 16, 21].

### Sequencing performances

A set of 90 FFPE samples, including 51 CRC and 39 NSCLC, was sequenced by NGS. Sequencing performance was assessed from the number and distribution of reads across the targeted regions. Among the 90 sequenced cases, 89 (98.9%) were successful (number of mapped reads >100000 or average base coverage >500x). The unsuccesfull case was considered non informative because of a number of reads <100 000 (40.072 reads) and average base coverage <500x (1.2X). Among the 89 successfully sequenced cases, one case was considered suboptimal (number of reads: 69.564 and average base coverage: 676x), however the quality of the sequencing was considered good enough for further analysis. All the other cases (n = 88, 97.8%) had a number of reads >100.000 and an average base coverage >1000X. The average number of reads per samples was 232.832 and the average base coverage depth was 2.296. On average 91.6% of the amplicons had a coverage depth of more than 500x (Table 3). There was no significant difference between CRC and NSCLC in terms of number of reads (p = 0.45), base coverage depth (p = 0.42) and percentage of amplicons with a coverage depth higher than 500x (p = 0.32) (Mann-Whitney test). For 12 cases, the amount of DNA obtained after extraction was too low to reach the required 10ng for targeted sequencing (DNA concentration ranging from 0.1 to 1.5ng/μl). The sequencing was successful for the 12 cases and no statistical difference was observed in terms of number of reads (p = 0.45), base coverage depth (p = 0.42) and in terms of percentage of amplicons with a coverage depth of more than 500x (p = 0.32) between samples with less than the required 10ng of DNA and samples with enough DNA (Mann-Whitney test, Table 3). This suggests that successful sequencing can be obtained from as little as 1 ng of DNA.

**Table 3:** Sequencing performances.

	Average number of reads	Average base coverage depth	% of amplicons > 500X
**Total (n = 89)**	232832	2296	91.6
**Tissue Type**			
Colon adenocarcinoma (n = 51)	238866	2361	92
Lung carcinoma (n = 38)	224735	2210	91.1
p-value	0.45	0.42	0.32
**Amount of DNA**			
≥10ng (n = 77)	232578	2291	92.8
<10ng (n = 12)	234466	2329	92.3
p-value	0.45	0.42	0.32
**Sample Type**			
Cell block (n = 9)	245976	2436	96.7
Biopsy (n = 23)	245945	2423	90.4
Surgical resection (n = 57)	225466	2223	93
p-value	0.5	0.45	0.02

We then considered the influence of different primary sample types on the sequencing performance. No statistical difference was observed in terms of number of reads and base coverage depth between surgical resections, biopsies and cell blocks (Kruskal-Wallis test, Table 3). However, the percentage of amplicons with a coverage depth of more than 500X was significantly higher for cell blocks than for biopsies (Kruskal-Wallis test p = 0.02 and post-hoc test p = 0.02).

The amplicons that showed a coverage below 250X were considered as non informative. This threshold was already used in the literature [22]. Some of the amplicons of the panel repeatedly failed to reach 250X (in more than 10% of the samples). These amplicons are listed in Table 4. The amplicons that repeatedly failed were the same for the different primary sample types (biopsies, cell blocks and surgical resection). For these amplicons, the GC content was significantly higher (average GC content in the 7 amplicons that repeatedly failed was 69.6% against 48% for the other amplicons, p = 0.00005), whereas the length was not significantly different (the average length of the 7 amplicons that repeatedly failed was 108 bp against 112 for the other amplicons).

**Table 4:** List of amplicons that consistently failed in >10% of samples to reach 250X coverage.

				Samples for which amplicon depth was < 250X	
Amplicon_ID	Gene	Exon	GC content (%)	n	%
CHP2_NOTCH1_1	*NOTCH1*	26	70.9	38	42.7
CHP2_STK11_4	*STK11*	6	60.4	35	39.3
CHP2_STK11_3	*STK11*	4–5	75.6	29	32.6
CHP2_FGFR3_2	*FGFR3*	9	67.9	21	23.6
CHP2_FGFR3_1	*FGFR3*	7	73.5	16	18.0
CHP2_FGFR3_5	*FGFR3*	18	72.8	14	15.7
CHP2_FGFR3_3	*FGFR3*	14	66	9	10.1

### Comparison with other methods

A set of 90 FFPE samples, including 51 CRC and 39 NSCLC, was sequenced by NGS. The mutational status of *KRAS* (exon 2) and *BRAF* (p.V600E) in CRC and *EGFR* (p.L858R and deletions in exon 19) in NSCLC had been previously assessed by PCR in the context of daily practice.

#### NSCLC

Sequencing was successful for 38 of the 39 samples tested (97%) whereas the *EGFR* analysis with the PCR method was successful for only 35 of the 39 samples (90%). 34 samples have successful testing both by NGS and PCR, allowing study of concordance.

Using NGS, mutations in *EGFR* were detected in 4/38 cases (10.5%). Three out of four *EGFR* mutations were also detected by PCR. For one sample that was considered as non informative by PCR, a p.L861Q *EGFR* mutation was detected using NGS (S2 Table). Moreover, a p.L858R *EGFR* mutation was detected by PCR and by NGS. However, for this sample the Variant Caller detected the mutation at an AF of 1.9%; given our criteria to consider a variant as authentic (see material and methods) we could not validate this variant.

Overall the concordance between the two methods for *EGFR* mutations detection was of 33/34 (97%).

**Fig 1:**
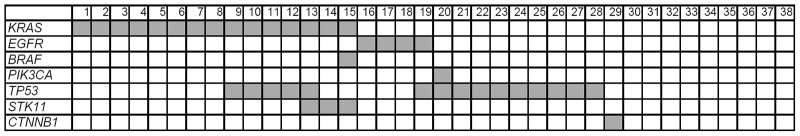
Mutational profiles of NSCLC. Mutations in different genes (rows) are indicated for each NSCLC sample (columns). A grey square indicates that a mutation (reported in the COSMIC database (http://www.sanger.ac.uk/cosmic), excluding polymorphism) was found with the Ampliseq Colon and Lung panel in the gene, whereas an empty square indicates that no relevant mutation was found for the gene.

Furthermore, mutations in other genes were detected by NGS: mutations in *KRAS* for 15/38 samples (39.5%), mutations in *TP53* for 15/38 patients (39.5%), mutations in *STK11* for 3/38 patients (7.9%), mutation in *BRAF* for one sample (2.6%), mutation in *PIK3CA* for one patient (2.6%), mutation in *CTNNB1* for one sample (2.6%). Mutational profiles of NSCLC were summarized in Fig 1 and in S2 Table. Overall, 29/38 samples were characterized by at least one mutation (76.3%).

The 2 KRAS mutations identified with an AF <6% (one p.G12S with an AF of 4%, one p.G12D with an AF of 5%) were verified by ddPCR. The 2 mutations were detected by ddPCR with an AF of 1.1 and 5.7%, respectively (S2 Table).

#### CRC

Sequencing and PCR were successful for all samples.

Using NGS, mutations in *KRAS* were detected in 30/51 cases (58.8%), whereas *KRAS* analysis with the PCR method detected only 23 cases of mutations in the *KRAS* gene (45.1%). Five of the 7 discordant cases were characterized by mutations in codons 59, 61 and 146 (exons 3 and 4) that were not covered by the PCR test. Mutations in codons 61 and 146 were tested and validated by ddPCR (S3 Table) For the 2 remaining cases not detected by PCR, *KRAS* p.G12V mutation was detected by NGS with an AF of 8 and 9%, respectively. These mutations were also detected by ddPCR with an AF of 12.5 and 9%, respectively. In the 23 concordant cases, AF of *KRAS* mutations were higher than 20% for 21 cases; only two cases were characterized by an AF of 9 and 10%, respectively.

The mutational status of *BRAF* by PCR was evaluated for 49 cases (for 2 cases there was not enough DNA). *BRAF* p.V600E mutation was detected for 5 patients (10.2%) using NGS or PCR. Moreover, a *BRAF* p.E586K mutation was detected using NGS.

Furthermore, mutations in other genes were detected by NGS: mutations in *NRAS* for 2/51 patients (3.9%), mutations in *TP53* for 32/51 patients (62.7%), mutations in *PIK3CA* for 10/51 patients (19.6%) and mutations in *FBXW7* for 5/51 samples (9.8%). The 2 NRAS mutations and the p.E545K, p.H1047 PIK3CA mutations were all validated by ddPCR (S3 Table).

**Fig 2:**
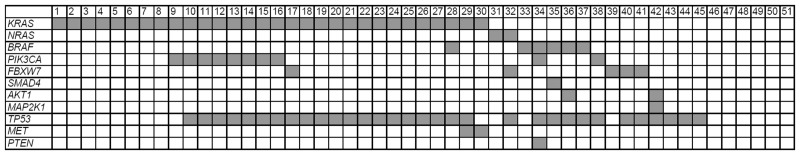
Mutational profiles of CRC. Mutations in different genes (rows) are indicated for each CRC sample (columns). A grey square indicates that a mutation (reported in the COSMIC database (http://www.sanger.ac.uk/cosmic), excluding polymorphism) was found with the Ampliseq Colon and Lung panel in the gene, whereas an empty square indicates that no relevant mutation was found for the gene.

Mutational profiles of CRC were summarised in Fig 2 and S3 Table. Overall, 45/51 samples were characterized by at least one mutation (88.2%).

## Discussion

A major advantage of NGS over traditional mutation detection methods is its ability to screen multiple mutations in multiple genes simultaneously without the need to perform several sequential tests. Several studies have already validated the use of NGS and its superiority in term of sensitivity, speed and cost compared to traditional methods. [18, 23, 24] In our own experience, for tests including more than two to three different hotspots, NGS is cheaper, faster and requires less DNA than would be needed for traditional methods. This is of particular importance for cytology samples and small-tissue biopsies for which several molecular alterations need to be screened, as for NSCLC samples e.g. [9, 18]. Indeed, NGS requires only 10 ng for the full colon and lung cancer panel while traditional methods can require up to 10 ng of DNA for each mutation tested.

The precision (reproducibility and repeatability) analysis using reference standards with a known AF allowed us to show that all mutations with an AF >5% were consistently detected. However, mutations with an AF <3% were detected inconsistently. In addition, when clinical samples were analyzed 5 times in 3 different experiments, the Variant Caller inconsistently detected mutations with an AF <3% that are not detected by ddPCR, suggesting that these mutations correspond to sequencing artefacts, as is often observed with DNA extracted from FFPE samples [25–27]. The 4% threshold was thus selected for mutation reporting as a balance between maximizing the sensitivity and minimizing the false-positive results due to technical artifact. This threshold is consistent with other sensitivity and specificity data reported in the literature for this NGS platform [16, 21]. Using this threshold, one case of NSCLC with a p.L858R EGFR mutation was missed. For this sample the Variant Caller detected the mutation at AF of 1.9%. However, given our criteria for considering a variant as authentic (AF >4% and variant coverage >30x), we could not validate this variant. It was recently proposed that known clinically relevant gene variants, such as *EGFR* mutations for NSCLC, should be reported irrespective of the AF [28]. In our current clinical practice, when we observe a known clinically relevant gene variant, but with an AF below the threshold, we report that the gene variant is suspected but not confirmed and that it would be interesting to test another sample from the patient if available.

Discrepancies between NGS and traditional methods were observed for 2 CRC cases with KRAS G12V mutations, both with an allelic frequency < 10%. This low AF can explain the discrepancies because the threshold of traditional method is varying from 3 to 20% of mutant DNA for the KRAS testing. For these 2 cases, the *KRAS* p.G12V mutations were validated by ddPCR.

One of the challenges using NGS is to interpret the detected mutations within the biological context. As already described [28], the variants can be grouped in three categories: i) those that may have a direct impact on patient care and are considered actionable; (ii) those that may have biological relevance but are not clearly actionable; and (iii) those that are of unknown significance. In the present study, NGS analysis detected mutations (other than *EGFR* mutations for NSCLC and than *KRAS* and *NRAS* for CRC) with potential clinical impact for 4 patients with NSCLC (one *PIK3CA* mutation and 3 *STK11* mutations) and for 14 patients with CRC (10 *PIK3CA* mutations, 5 *BRAF* mutations—one patient harbouring *PIK3CA* and *BRAF* mutations). Indeed, preclinical data support the argument that NSCLC cell lines with *PIK3CA* or *STK11* and *KRAS* mutation show increased sensitivity to PIK3 inhibitors or MAPK and mTOR signalling inhibition, respectively [29]-[30]. Moreover, a phase I dose-escalation clinical trial of a pan-class I PI3K inhibitor in patients with advanced solid tumors—primarily colorectal, breast, and lung—showed preliminary antitumor activity [31]. In the same way, apparent anti-tumour activity was observed for patients with *BRAF* mutated CRC treated with a selective mutant BRAF inhibitor [32].

In conclusion, the present study validated the clinical applicability of the Ion Ampliseq Colon and Lung cancer panel on the Ion Torrent Personal Genome Machine for screening lung and colorectal cancers. Overall, the AmpliSeq colon/lung cancer panel was specific and sensitive enough for mutation analysis of gene panels and can be incorporated into clinical daily practice.

## Supporting Information

S1 File Supplementary methods: detection of KRAS, BRAF and EGFR mutations and droplet digital PCR. (DOCX)

S1 Table Coverage analysis for variants not consistently detected in the precision analysis. (DOC)

S2 Table Sequencing results of NSCLC. (DOCX)

S3 Table Sequencing results of CRC. (DOCX)
