# Supplementary material for: Clinical Validation of Targeted Next Generation Sequencing for Colon and Lung Cancers
Source: PLoS One. 2015 Sep 14;10(9):e0138245. doi: 10.1371/journal.pone.0138245 (PMC4569137; doi:10.1371/journal.pone.0138245)
Supplement: S1 File — (DOCX) [file pone.0138245.s001.docx]

**S1 File : Supplementary methods : detection of KRAS, BRAF and EGFR mutations and droplet digital PCR**

***BRAF* mutation detection**

The status of *BRAF* p.V600E mutation was determined in 49 of the 51 colorectal adenocarcinomas by allele specific PCR as described by Jarry et al. [1]. Briefly, two forward primers with variations in their 3’ nucleotides specific to the wild type (V; AGGTGATTTTGGTCTAGCTACAGT) or the mutated variant (E; AGGTGATTTTGGTCTAGCTACAGA), and one common reverse primer (AS; TAGTAACTCAGCAG CATCTCAGGGC) were used. The sequence-specific forward and the reverse primers were then combined for each sample in ‘PCR V’ (primers V and AS), and ‘PCR E’ (primers E and AS). PCR amplifications were performed on a LightCycler® 2.0 using the LightCycler FastStart DNA Master SYBR Green I (Roche) and starting from 20 ng of DNA. Samples were considered positive for *BRAF* p.V600E mutation when ΔCp = CpPCR E – CpPCR V < 3.15. This threshold was set as the mean ΔCp – 3 x the standard deviations of 30 non tumor tissues (data not shown). The limit of detection of the test, determined by serial dilution of DNA of a *BRAF* p.V600E positive cell line (HT-29) in DNA from a *BRAF* p.V600E negative cell line (EA.hy926), is 10% of mutant DNA.

***KRAS* mutation detection**

The status of KRAS mutations located in the codon 12 (n=6) and 13 (n=1) were determined by an allelic discrimination assay, as previously described [2].

***EGFR* mutation detection**

p.T790M and p.L858R mutations were detected by using a methodological approach adapted from a previously published paper [3].

Briefly (Figure A), we used a combination of a LNA oligonucleotide designed to limit as much as possible amplification of the WT allele in order to enrich the DNA preparation in mutant allele when present; this latter being detected by the use of a mutant specific hydrolysis probe.

One control PCR has also been set up for each of these two mutation detection systems by using the same amplification primers. However, the LNA and hydrolysis probe were replaced by SYTO82, an intercalating agent able to detect double-strand DNA. The aim of this test was to confirm the qualitative amplifiability of the DNA.

The limit of detection of the test was determined by serial dilution of a linearized plasmid containing the mutant allele with genomic DNA from a healthy individual.

A cut-off value of positivity has been determined by testing at least 16 « negative » samples. A Cq value has been recorded for each sample presenting an amplification plot.

A 99% confidence interval has been determined (Average Cq value – 3 S.D.) and each sample harboring a Cq value below that cut-off has been scored as « positive » for the mutation.

Considering our validation pipeline, the limit of detection has been fixed at 5% for the p.T790M and 0.5% for the p.L858R.

Regarding the exon19 deletions mutations, set up process was very similar to the one described above except that we used SYTO82 to detect mutations instead of specific probes. Indeed, many different deletions being described into a hot-spot region, it would have been difficult to design as many probes as they are different type of deletions. Therefore, our methodological approach allowed us to detect the presence of a deletion but not to determine which type of deletion we were dealing with. Amplification primers were also combined with a LNA oligonucleotide in order to limit amplification of the WT allele. In presence of a mutant allele, the LNA oligonucleotide will not hybridize and mutated amplicons will be generated and detected with the help of SYTO82.

The same procedure as described above has been followed to determine the cut-off value of positivity and the limit of detection. This latter has been determined as being 1%.

In order to confirm the specificity of the PCR signal obtained for exon19 deletion mutations, each positive sample was checked on an agarose gel in order to rule out the presence of primer dimers or other artifacts which could generate weak false positive signals.

Primer sequences, LNA and probes are recorded in Table A

PCR cycle conditions, PCR instrument and working concentration for each component are described in Table B.

**droplet digital PCR**

ddPCR was performed using the Bio-Rad QX-200 system (Biorad, Hercules, USA). Assays were purchased from Bio-Rad at 20x concentration (see list below).

| mutation | Mutation Assay | Reference assay |
| --- | --- | --- |
| KRAS p.A59T | dHsaIS2505768 | dHsaIS2505769 |
| KRAS p.G12C | dHsaCP2000007 | dHsaCP2000008 |
| KRAS p.G12S | dHsaCP2000011 | dHsaCP2000012 |
| KRAS p.G12V | dHsaCP2000005 | dHsaCP2000006 |
| KRAS p.Q61H | [dHsaCP2000133](http://www.bio-rad.com/en-us/prime-pcr-assays/assay/dhsacp2000133-primepcr-ddpcr-mutation-assay-kras-p-q61h-human) | [dHsaCP200013](http://www.bio-rad.com/en-us/prime-pcr-assays/assay/dhsacp2000133-primepcr-ddpcr-mutation-assay-kras-p-q61h-human)4 |
| KRAS p.Q61R | dHsaCP2000135 | dHsaCP2000136 |
| KRAS p.A146T | dHsaCP2000079 | dHsaCP2000080 |
| KRAS p.G12D | dHsaCP2000001 | dHsaCP2000002 |
| NRAS p.G12D | dHsaCP2000095 | dHsaCP2000096 |
| NRAS p.Q61K | dHsaCP2000067 | dHsaCP2000068 |
| PIK3CA p.E545K | dHsaCP2000075 | dHsaCP2000076 |
| PIK3CA p.H1047L | dHsaCP2000123 | dHsaCP2000124 |
| PIK3CA p.H1047Q | dHsaIS2506156 | dHsaIS2506157 |
| TP53 p.H168Y | dHsaIS2500720 | dHsaIS2500721 |
| TP53 p.R181C | dHsaIS2501892 | dHsaIS2501893 |

ddPCR reaction mixtures contained a final concentration of 250nM for each of the probes, 450nM for the forward and reverse primers, 1x ddPCR^TM^ Supermix for Probes (No dUTP) (Bio-Rad #186-3024 USA) and 24ng of genomic DNA in a final volume of 24 µl. Twenty µl of this ddPCR reaction volume were loaded in appropriate wells of a DG8 cartridge (Bio-Rad #186-4008, USA) with 70 µl of generator oil (Bio-Rad #186-3030, USA) in to the oil well. Samples are partitioned into approximately 20,000 water-oil emulsion droplets, each 0.85 nanoliter in volume, using the QX200™ Droplet generator™ (Bio-Rad). Forty µl of this water-oil emulsion were used for the ddPCR assay by transferring it into a 96-wells plate sealed with a PX1 ™ PCR plate Sealer (Bio-Rad, USA). ddPCR were performed with a T100™ thermal cycler (Bio-Rad, USA) under the following conditions: 1 cycle of 95°C for 10 min, 40 cycles of 94°C for 30s and 55°C for 1 min, and 1 cycle of 98°C for 10 min. Cycled droplets were read individually with the QX200TM droplet reader (Bio-Rad). No template control wells (NTC) were included in the assays. Data was analyzed using QuantaSoft TM software version 1.6.6.0320.

**Figure A**:


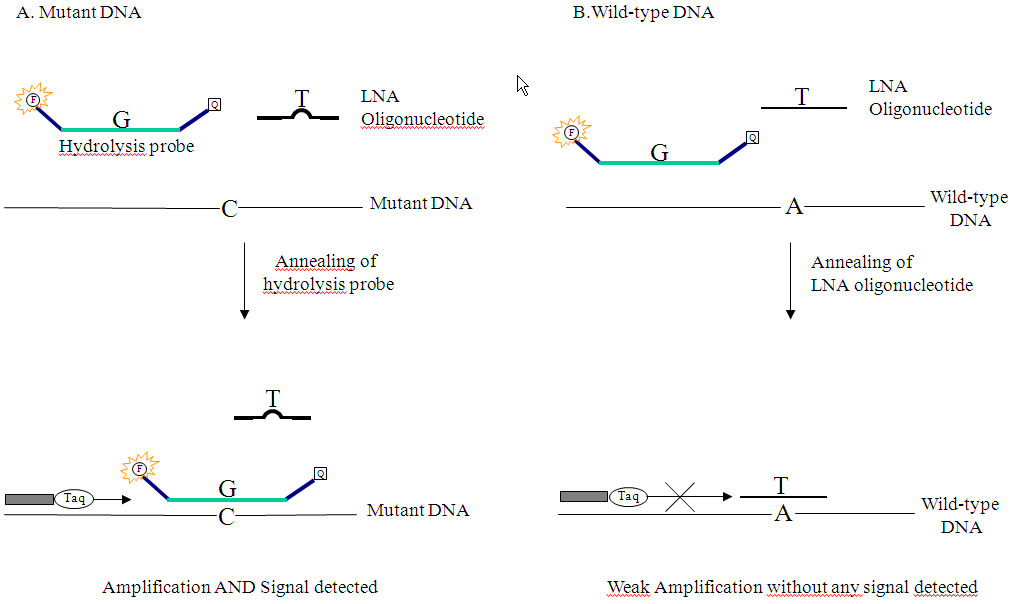


Adapted from Sidon et al. [3]. Overview of our methodological approach*. A*. The perfect matching between the hydrolysis probe and the mutated DNA allows annealing of the probe to the target DNA sequence and detection of this latter. At the opposite, the single base pair mismatch between the LNA blocking sequence and the mutated sequence prevents annealing of this modified nucleotide. *B*. The LNA oligonucleotide anneals to the wild-type target sequence thereby preventing amplification of this target. The LNA oligonucleotide allows thus to amplify preferably the mutated sequence. No hybridization of hydrolysis probe to the wild-type DNA occurs due to the base pair mismatch between both sequences.

**Table A : sequences of primers, LNA and probes**

| **Exon 19 (del)** |  |  |
| --- | --- | --- |
| LNA blocking oligonucleotide | EGFREx19LNA1 | {GAATTAAGAGAAGCA} |
| Forward primer | EGFREx19F | CTGGATCCCAGAAGGTGAGA |
| Reverse primer | EGFREx19R | ATCGAGGATTTCCTTGTTGG |
| **Exon 20 (p.T790M)** |  |  |
| LNA blocking oligonucleotide | EGFRT790MLNA | {TCATCACGCAGC} |
| Forward primer | EGFRT790MD1 | GCATCTGCCTCACCTCCAC |
| Reverse primer | EGFRT790MR1 | GTCTTTGTGTTCCCGGACAT |
| Mutation detection probe | EGFRT790M Probe | FAM-CTCATC[4]CAGCTCATGCC-BHQ1 |
| **Exon 21 (p.L858R)** |  |  |
| LNA blocking oligonucleotide | EGFR21LNA | {TGGGLTGG} |
| Forward primer | EGFRL858RF | AGCCAGGAACGTACTGGTGA |
| Reverse primer | EGFRL858RR | TGCCTCCTTCTGCATGGTAT |
| Mutation detection probe | L858R Probe | FAM-GGGC{GG}GCCA-BHQ1 |

Letters between brackets correspond to LNA type nucleotides

**Table B : PCR cycle conditions, PCR instrument and working concentration for each component**

|  | **Del19 mutation detection** | | **p.T790M mutation detection** | | **p.L858R mutation detection** | |
| --- | --- | --- | --- | --- | --- | --- |
|  | **"Control" PCR** | **"Mutant" PCR** | **"Control" PCR** | **"Mutant" PCR** | **"Control" PCR** | **"Mutant" PCR** |
|  | Concentration | Concentration | Concentration | Concentration | Concentration | Concentration |
| Taqman Fast Universal PCR Master Mix, No AmpErase (Applied Biosystems ref. 4366073) | 1x | 1x | 1x | 1x | 1x | 1x |
| Forward primer | 200nM | 200nM | 200nM | 200nM | 200nM | 200nM |
| Reverse primer | 200nM | 200nM | 200nM | 200nM | 200nM | 200nM |
| SYTO82 | 2µM | 2µM | 2µM | / | 2µM | / |
| Mutation detection probe | / | 200nM | / | 100nM | / | 300nM |
| LNA blocking oligonucleotide | / | / | / | 50nM | / | 100nM |
|  |  |  |  |  |  |  |
| Genomic DNA | 10ng | 10ng | 10ng | 10ng | 10ng | 10ng |
|  |  |  |  |  |  |  |
| PCR reaction volume | 20µL | 20µL | 20µL | 20µL | 20µL | 20µL |

| **PCR intrument ABI 7500 FAST** | |  |  |  |  |  |  |
| --- | --- | --- | --- | --- | --- | --- | --- |
| Thermal Profile (Del19 and p.L858R) | | | |  | Settings | | |
| Stage | Repetitions | Temperature | Time (min.) | Ramp Rate | PCR Volume (µl) | Run Mode | Data Collection |
| 1 | 1 | 95.0 °C | 0:20 | Auto | 20 | Fast 7500 | Stage 2 Step 2 (60 @ 0:30) |
| 2 | 40 | 95.0 °C | 0:03 | Auto |  |  |  |
|  |  | 60.0 °C | 0:30 | Auto |  |  |  |
| 3 (Dissociation) | 1 | 95.0 °C | 0:15 | Auto |  |  |  |
|  |  | 60.0 °C | 1:00 | Auto |  |  |  |
|  |  | 95.0 °C | 0:15 | Auto |  |  |  |
|  |  |  |  |  |  |  |  |
| Thermal Profile (p.T790M) | | | |  | Settings | | |
| Stage | Repetitions | Temperature | Time (min.) | Ramp Rate | PCR Volume (µl) | Run Mode | Data Collection |
| 1 | 1 | 95.0 °C | 0:20 | Auto | 20 | Fast 7500 | Stage 2 Step 2 (60 @ 0:30) |
| 2 | 40 | 95.0 °C | 0:03 | Auto |  |  |  |
|  |  | 65.0 °C | 0:30 | Auto |  |  |  |
| 3  (Dissociation) | 1 | 95.0 °C | 0:15 | Auto |  |  |  |
|  |  | 60.0 °C | 1:00 | Auto |  |  |  |
|  |  | 95.0 °C | 0:15 | Auto |  |  |  |

**Supplementary References :**

1. Jarry A, Masson D, Cassagnau E, Parois S, Laboisse C, et al. Real-time allele-specific amplification for sensitive detection of the BRAF mutation V600E. Mol Cell Probes. 2004;18: 349-352.

2. De Roock W, Piessevaux H, De Schutter J, Janssens M, De Hertogh G, et al. KRAS wild-type state predicts survival and is associated to early radiological response in metastatic colorectal cancer treated with cetuximab. Ann Oncol. 2008;19: 508-515.

3. Sidon P, Heimann P, Lambert F, Dessars B, Robin V, et al. Combined locked nucleic acid and molecular beacon technologies for sensitive detection of the JAK2V617F somatic single-base sequence variant. Clin Chem. 2006; 52: 1436-1438.

4. Tsai J, Lee JT, Wang W, Zhang J, Cho H, et al. Discovery of a selective inhibitor of oncogenic B-Raf kinase with potent antimelanoma activity. Proc Natl Acad Sci U S A. 2008; 105: 3041-3046.
