# Supplementary material for: Clinical Validation of Targeted Next Generation Sequencing for Colon and Lung Cancers
Source: PLoS One. 2015 Sep 14;10(9):e0138245. doi: 10.1371/journal.pone.0138245 (PMC4569137; doi:10.1371/journal.pone.0138245)
Supplement: S1 Table — (DOC) [file pone.0138245.s002.doc]

**S1** Table : Coverage analysis for variants not consistently detected in the precision analysis

|  |  | **NGS results : variant coverage/total coverage (AF)** | | | | |  |
| --- | --- | --- | --- | --- | --- | --- | --- |
| **Sample** | **Mutations** | **Rep 1** | **Rep 2** | **Rep 3** | **Rep 4** | **Rep 5** | **Mean AF (SD)** |
| **1** | *TP53* p.R181C .  *TP53* p.H168Y .  *TP53* p.R213Q | 7/384 (1.8%;D)  1/388 (0.3%;ND)  0/926 (0%;ND) | 0/198 (0%;ND)  0/197 (0%;ND)  12/599 (2%;D) | 0/196 (0%;ND)  0/194 (0%;ND)  0/362 (0%;ND) | 0/266 (0%;ND)  5/274 (1.8%;D)  1/602 (0.2%;ND) | 0/489 (0%;ND)  1/494 (0.2%;ND)  0/798 (0%;ND) | 0.4% (0.8) .  0.4% (0.8) .  0.4% (0.9) |
| **2** | *NOTCH1* p.V1758delV .  *TP53* p.H178N | 4/702 (0.6%;ND)  0/1526 (0%;ND) | 6/925 (0.7%;ND)  1/1885 (0.05%;ND) | 1/130 (0.8%;ND)  0/195 (0%;ND) | 7/409 (1.7%;D)  0/873 (0%;ND) | 6/650 (0.9%;ND)  34/1730 (2%;D) | 0.9 % (0.4) .  0.4 % (0.9) |
| **Multiplex reference standard** | *EGFR* p.E746-A750delELREA *(2.0%)*  *EGFR* p.L858R *(2.7%) .*  *EGFR* p.T790M *(0.9%)* | 37/1845 (2%;D)  24/2052 (1.1%;ND)  13/1616 (0.8%;ND) | 34/2209 (1.5%;ND)  34/2254 (1.5%;ND)  27/2076 (1.3%;ND) | 9/610 (1.5%;ND)  16/940 (1.7%;D)  7/473 (1.5%;ND) | 49/1978 (2.4%;D)  24/1961 (1.2%;ND)  15/1494 (1%;ND) | 19/2352 (0.8%;ND)  25/1633 (1.5%;D)  7/1613 (0.4%;ND) | 1.6% (0.6) .  1.4 % (0.2) .  1 %(0.4) |

AF : allelic frequency ; D: variant detected by the variant caller ; ND : variant detected by the variant caller; SD : standard deviation
