# Supplementary material for: Clinical Validation of Targeted Next Generation Sequencing for Colon and Lung Cancers
Source: PLoS One. 2015 Sep 14;10(9):e0138245. doi: 10.1371/journal.pone.0138245 (PMC4569137; doi:10.1371/journal.pone.0138245)
Supplement: S2 Table — (DOCX) [file pone.0138245.s003.docx]

**S2 Table : Sequencing results of NSCLC**

| **Sample** | **% tumor cells** | ***EGFR* PCR results** | **NGS Results** | | | | | | | | | | | | | **ddPCR Results** |
| --- | --- | --- | --- | --- | --- | --- | --- | --- | --- | --- | --- | --- | --- | --- | --- | --- |
|  |  |  | ***EGFR*** | | ***KRAS*** | | ***BRAF*** | | ***PIK3CA*** | | ***CTNNB1*** | ***TP53*** | ***STK11*** | ***MET*** | ***FGFR3*** |  |
| 1 | 100 | deletion exon 19 | p.E746_A750delELREA (16%) | |  | |  | |  | |  |  |  |  |  |  |
| 2 | 50 | deletion exon 19 | p.L747_T751delREAT (19%) | |  | |  | |  | |  | p.H178D (17.7%) |  |  |  |  |
| 3 | 50 | p.L858R | p.L858R (9.5%) | |  | |  | |  | |  |  |  |  |  |  |
| 4 | 50 | Failed | p.L861Q (32.4%) | |  | |  | |  | |  |  |  |  |  |  |
| 5 | 10 | p.L858R |  | |  | |  | |  | |  |  |  |  |  |  |
| 6 | 20 | Failed |  | | p.G12A (39.9%) | |  | |  | |  |  |  |  |  |  |
| 7 | 75 | - |  | | p.G12C (30.7%) | |  | |  | |  | p.W91* (24.5%) |  |  |  |  |
| 8 | 50 | - |  | | p.G12C (23.3%) | |  | |  | |  |  |  |  |  |  |
| 9 | 80 | - |  | | p.G12C (51.7%) | |  | |  | |  |  |  | p.N375S (93.3%) |  |  |
| 10 | 10 | - |  | | p.G12C (43.7%) | |  | |  | |  | p.R156P (31.4%) |  |  |  |  |
| 11 | 90 | - |  | | p.G12C (14.1%) | |  | |  | |  |  |  | p.N375S (49.0%) |  |  |
| 12 | 80 | - |  | | p.G12C (52.0%) | |  | |  | |  | p.V274F (41.5%) |  |  |  |  |
| 13 | <10 | - |  | | p.G12D (16.7%) | |  | |  | |  |  | p.D194Y (15.0%) |  |  |  |
| 14 | 70 | - |  | | p.G12D (5.1%) | |  | |  | |  |  |  |  | p.F384L (47.0%) | KRAS p.G12D 5.7% |
| 15 | 90 | - |  | | p.G12S (4.0%) | |  | |  | |  | p.R280I (62.5%) | p.E199* (72.9%) |  |  | KRAS p.G12S 1.1% |
| 16 | 90 | - |  | | p.G12S (64.5%) | |  | |  | |  |  |  |  |  |  |
| 17 | 50 | - |  | | p.G12V (47.1%) | |  | |  | |  |  |  |  |  |  |
| 18 | 50 | - |  | | p.G13C (9.5%) | | p.D594G (15.2%) | |  | |  |  | p.D194Y (20.9%) |  |  |  |
| 19 | 60 | - |  | | p.G13C (40.8%) | |  | |  | |  | p.R273H (18.0%) |  |  |  |  |
| 20 | 90 | - |  | | p.Q61H (18.9%) | |  | |  | |  |  |  |  |  | KRAS p.Q61H 20.2% |
| 21 | 60 | - |  | |  | |  | | p.E542K (42.5%) | |  | p.R280S (33.2%) |  |  | p.F384L (85.9%) |  |
| 22 | 70 | - |  | |  | |  | |  | | p.S33F (10.3%) |  |  |  |  |  |
| **Sample** | **% tumor cells** | ***EGFR* PCR results** | **NGS Results** | | | | | | | | | | | | | **ddPCR Results** |
|  |  |  | ***EGFR*** | ***KRAS*** | | ***BRAF*** | | ***PIK3CA*** | | ***CTNNB1*** | | ***TP53*** | ***STK11*** | ***MET*** | ***FGFR3*** |  |
| 23 | <10 | Failed |  |  | |  | |  | |  | | p.G266E (10.9%) |  |  |  |  |
| 24 | 10 | - |  |  | |  | |  | |  | | p.K164E (8.0%) |  |  |  |  |
| 25 | 10 | - |  |  | |  | |  | |  | | p.R267P (12.0%) |  |  |  |  |
| 26 | 50 | - |  |  | |  | |  | |  | | p.R249S (12.7%) |  |  |  |  |
| 27 | 50 | - |  |  | |  | |  | |  | | p.E294* (16.0%) |  |  |  |  |
| 28 | 80 | - |  |  | |  | |  | |  | | p.C238F (46.2%) |  | p.N375S (47.3%) |  |  |
| 29 | 50 | - |  |  | |  | |  | |  | | p.H179Y (50.3%) |  |  |  |  |
| 30 | 40 | - |  |  | |  | |  | |  | | p.E285K (12.3%) |  |  |  |  |
| 31 | 70 | - |  |  | |  | |  | |  | |  |  | p.N375S (47.4%) |  |  |
| 32 | <10 | Failed |  |  | |  | |  | |  | |  |  |  |  |  |
| 33 | 70 | - |  |  | |  | |  | |  | |  |  |  |  |  |
| 34 | 90 | - |  |  | |  | |  | |  | |  |  |  |  |  |
| 35 | 50 | - |  |  | |  | |  | |  | |  |  |  |  |  |
| 36 | 50 | - |  |  | |  | |  | |  | |  |  |  |  |  |
| 37 | 90 | - |  |  | |  | |  | |  | |  |  |  |  |  |
| 38 | 90 | - |  |  | |  | |  | |  | |  |  |  |  |  |
| **Total** |  |  | **4/38** | **15/38** | | **1/38** | | **1/38** | | **1/38** | | **15/38** | **3/38** | **4/38** | **2/38** |  |
| **%** |  |  | **10.5%** | **39.5%** | | **2.6%** | | **2.6%** | | **2.6%** | | **39.5%** | **7.9%** | **10.5%** | **5.3%** |  |
