# Supplementary material for: Clinical Validation of Targeted Next Generation Sequencing for Colon and Lung Cancers
Source: PLoS One. 2015 Sep 14;10(9):e0138245. doi: 10.1371/journal.pone.0138245 (PMC4569137; doi:10.1371/journal.pone.0138245)
Supplement: S3 Table — (DOCX) [file pone.0138245.s004.docx]

**S3 Table : Sequencing results of CRC**

| **Sample** | **% tumor cells** | ***KRAS* PCR results** | ***BRAF* PCR results** | **NGS Results** | | | | | | | | | **ddPCR results** |
| --- | --- | --- | --- | --- | --- | --- | --- | --- | --- | --- | --- | --- | --- |
|  |  |  |  | ***KRAS*** | ***BRAF*** | ***NRAS*** | ***PIK3CA*** | ***FBXW7*** | ***TP53*** | ***PTEN*** | ***MET*** | **other** |  |
| 1 | 25 | - |  | p.A146T (34.6%) |  |  |  |  | p.S215N (5.3%)  p.R196* (46.85%) |  |  |  | KRAS p.A146T 37.0% |
| 2 | 50 | - |  | p.A146T (67.8%) |  |  |  |  | p.R306* (51.2%) |  |  |  | KRAS p.A146T 66.1% |
| 3 | 60 | - |  | p.A59E (42.1%) |  |  |  |  |  |  |  |  |  |
| 4 | 40 | p.G12A |  | p.G12A (32.3%) |  |  | p.E542K (17.8%) |  |  |  |  |  |  |
| 5 | 30 | p.G12A |  | p.G12A (26.6%) |  |  | p.E545K (26.3%) |  | p.R282W (31.7%) |  |  |  | PIK3CA p.E545K 26.1% |
| 6 | 50 | p.G12A |  | p.G12A (23.2%) |  |  |  |  | p.Y163C (37.05%) |  |  |  |  |
| 7 | 60 | p.G12C |  | p.G12C (46.9%) |  |  |  |  |  |  |  |  |  |
| 8 | 40 | p.G12D |  | p.G12D (41.2%) |  |  |  |  | p.A159fs*11 (42.5%) |  | p.N375S (51%) |  |  |
| 9 | 60 | p.G12D |  | p.G12D (41.2%) |  |  | p.E542K (38.1%) |  | p.V272M (67%) |  |  |  |  |
| 10 | 40 | p.G12D |  | p.G12D (33.7%) |  |  |  |  | p.R175H (40.1%) |  |  |  |  |
| 11 | 80 | p.G12D |  | p.G12D (49.5%) |  |  | p.E542K (31.9%) |  | p.G266V (33.9%) |  |  |  |  |
| 12 | 50 | p.G12S |  | p.G12S (25%) |  |  |  | p.R505C (21.6%) | p.R196* (30%) |  |  |  |  |
| 13 | 60 | p.G12S |  | p.G12S (42.7%) |  |  |  |  | p.R273C (58.2%) |  |  |  |  |
| 14 | 30 | - |  | p.G12V (7.6%) |  |  |  |  |  |  |  |  | KRAS p.G12V 12.5% |
| 15 | 50 | p.G12V |  | p.G12V (33.8%) |  |  |  |  | p.C242F (73.1%) |  |  |  |  |
| 16 | 90 | p.G12V |  | p.G12V (57.7%) |  |  |  |  | p.R175H (64%) |  |  |  |  |
| 17 | 20 | - |  | p.G12V (9.1%) |  |  | p.E545K (6.7%) |  | p.W91* (7.8%) |  |  |  | KRAS p.G12V 9.1%  PIK3CA p.E545K 9% |
| 18 | 60 | p.G12V |  | p.G12V (27.8%) |  |  | p.E545K (5.9%) |  | p.R273H (33.7%)  p.C229fs*10 (4.5%) |  |  |  | PIK3CA p.E545K 6.2% |
| 19 | 30 | p.G12V |  | p.G12V (45.3%) |  |  |  |  | p.R273H (36.3%) |  |  |  |  |
|  |  |  |  |  |  |  |  |  |  |  |  |  |  |
| **sample** | **% tumor cells** | **KRAS PCR result** | **BRAF PCR result** | **NGS Results** | | | | | | | | | **ddPCR Results** |
|  |  |  |  | **KRAS** | **BRAF** | **NRAS** | **PIK3CA** | **FBXW7** | **TP53** | **PTEN** | **MET** | **other** |  |
|  |  |  |  |  |  |  |  |  |  |  |  |  |  |
| 20 | 70 | p.G12V |  | p.G12V (55.7%) |  |  |  |  | p.Y220C (81.5%) |  |  |  |  |
| 21 | 40 | p.G12V |  | p.G12V (34%) |  |  |  |  |  |  |  |  |  |
| 22 | 70 | p.G12V |  | p.G12V (87.9%) |  |  |  |  |  |  | p.E166D (49%° |  |  |
| 23 | 70 | p.G12V |  | p.G12V (39.6%) |  |  |  |  |  |  |  |  |  |
| 24 | 25 | p.G13D |  | p.G13D (10.4%) |  |  |  |  |  |  |  |  |  |
| 25 | 20 | p.G13D |  | p.G13D (9.3%) |  |  | p.E545K (8.8%) |  | p.G245V (10.5%) |  |  |  | PIK3CA p.E545K 8.8% |
| 26 | 30 | p.G13D |  | p.G13D (46.5%) |  |  |  |  | p.R248Q (37.9%) |  |  |  |  |
| 27 | 60 | p.G13D |  | p.G13D (43.2%) |  |  |  |  |  |  |  |  |  |
| 28 | 40 | p.G13D |  | p.G13D (42.4%) |  |  | p.E542K (38%) |  | p.R110L (54.9%) |  |  |  |  |
| 29 | 10 |  | Not enough DNA | p.Q61H (5%) |  |  |  |  |  |  |  |  | Not enough DNA |
| 30 | 60 |  |  | p.Q61R (41.2%) | p.E586K (28.3%) |  |  |  | p.G245S (60.2%) |  |  |  | KRAS p.Q61R 46.1% |
| 31 | 50 |  | Not enough DNA |  |  |  |  | p.S582L (23.1%)  p.R393* (22%° | p.R196* (40.6%) |  |  |  |  |
| 32 | 25 |  | p.V600E |  | p.V600E (23.1%) |  | p.H1047Q (11.1%) |  | p.R4273C (12.8%) | p.E288fs*3 (9.3%) |  |  | PIK3CA p.H1047Q 9.1% |
| 33 | 50 |  | p.V600E |  | p.V600E (34%) |  |  |  | p.T253A (18.1%) |  |  | SMAD4 p.R361H (49.6%) |  |
| 34 | 50 |  | p.V600E |  | p.V600E (31.4%) |  |  |  |  |  |  |  |  |
| 35 | 80 |  | p.V600E |  | p.V600E (38.8%) |  |  |  | p.E180K (38.5%)  p.S240G (22.3%° |  |  | AKT1 p. E17K (29.6%) |  |
| 36 | 50 |  | p.V600E |  | p.V600E (39.5%) |  |  |  | p.R213* (76.6%) |  |  |  |  |
| 37 | 20 |  |  |  |  | p.G12D (21.8%) |  | p.R385C (21.4%) | p.Y220H (26.3%) |  |  |  | NRAS p.G12D 22.3% |
|  |  |  |  |  |  |  |  |  |  |  |  |  |  |
| **sample** | **% tumor cells** | **KRAS PCR result** | **BRAF PCR result** | **NGS Results** | | | | | | | | | **ddPCR results** |
|  |  |  |  | **KRAS** | **BRAF** | **NRAS** | **PIK3CA** | **FBXW7** | **TP53** | **PTEN** | **MET** | **other** |  |
| 38 | 40 |  |  |  |  | p.Q61K (12%) |  |  |  |  |  |  | NRAS p.Q61K 10.2% |
| 39 | 60 |  |  |  |  |  |  |  | p.R273C (56.5%) |  |  | AKT1 p.E17K (48.2%)  MAP2K1 p.K57N (77%) |  |
|  |  |  |  |  |  |  |  |  |  |  |  |  |  |
| 40 | <10 |  |  |  |  |  |  |  | p.R196* (63.7%) |  |  |  |  |
| 41 | 50 |  |  |  |  |  | p.H1047L (31.7%) |  | p.S241F (70.5%) |  |  |  | PIK3CA p.H1047L 31.5% |
| 42 | 30 |  |  |  |  |  |  |  | p.R273C (47.4%) |  |  |  |  |
| 43 | 60 |  |  |  |  |  |  | p.R385C (74.2%) | p.R282W (66.8%) |  |  |  |  |
| 44 | 50 |  |  |  |  |  |  |  |  |  |  |  |  |
| 45 | 25 |  |  |  |  |  |  | p.R385C |  |  |  |  |  |
| 46 | 50 |  |  |  |  |  |  |  |  |  |  |  |  |
| 47 | <10 |  |  |  |  |  |  |  |  |  |  |  |  |
| 48 | 40 |  |  |  |  |  |  |  |  |  |  |  |  |
| 49 | 40 |  |  |  |  |  |  |  | R175H (25%) |  |  |  |  |
| 50 | 40 |  |  |  |  |  |  |  |  |  |  |  |  |
| 51 | 80 |  |  |  |  |  |  |  |  |  |  |  |  |
| **Total** |  |  |  | **30/51** | **6/51** | **2/51** | **10/51** | **5/51** | **32/51** | **1/51** | **2/51** |  |  |
| **%** |  |  |  | **58.8%** | **11.8%** | **3.9%** | **19.6%** | **9.8%** | **62.7%** | **2%** | **3.9%** |  |  |
